# Impaired Hepatic Adaptation to Chronic Cholestasis induced by Primary Sclerosing Cholangitis

**DOI:** 10.1038/srep39573

**Published:** 2016-12-23

**Authors:** Malgorzata Milkiewicz, Marta Klak, Agnieszka Kempinska-Podhorodecka, Anna Wiechowska-Kozlowska, Elzbieta Urasinska, Malgorzata Blatkiewicz, Ewa Wunsch, Elwyn Elias, Piotr Milkiewicz

**Affiliations:** 1 Department of Medical Biology, Pomeranian Medical University, Szczecin, Poland; 2 Endoscopy Unit, Ministry of Internal Affairs Hospital, Szczecin, Poland; 3 Department of Pathology, Pomeranian Medical University, Szczecin, Poland; 4 Translational Medicine Group, Pomeranian Medical University, Szczecin, Poland; 5 Liver and Hepatobiliary Unit, University of Birmingham, Birmingham, UK; 6 Liver and Internal Medicine Unit, Department of General, Transplant and Liver Surgery of the Medical University of Warsaw, Warsaw, Poland

## Abstract

Pathogenesis of primary sclerosing cholangitis (PSC) may involve impaired bile acid (BA) homeostasis. We analyzed expressions of factors mediating enterohepatic circulation of BA using ileal and colonic (ascending and sigmoid) biopsies obtained from patients with PSC with and without ulcerative colitis (UC) and explanted PSC livers. Two-fold increase of BA-activated farnesoid X receptor (FXR) protein levels were seen in ascending and sigmoid colon of PSC patients with correspondingly decreased apical sodium-dependent BA transporter (ASBT) gene expression. This was associated with increased OSTβ protein levels in each part of analyzed gut. An intestinal fibroblast growth factor (FGF19) protein expression was significantly enhanced in ascending colon. Despite increased hepatic nuclear receptors (FXR, CAR, SHP), and FGF19, neither CYP7A1 suppression nor CYP3A4 induction were observed. The lack of negative regulation of BA synthesis may be accountable for lower levels of cholesterol observed in PSC in comparison to primary biliary cholangitis (PBC). In conclusion, chronic cholestasis in PSC induces adaptive changes in expression of BA transporters and FXR in the intestine. However hepatic impairment of expected in chronic cholestasis downregulation of CYP7A1 and upregulation of CYP3A4 may promote BA-induced liver injury in PSC.

Primary sclerosing cholangitis (PSC) is a chronic cholestatic liver condition that affects both small and large bile ducts. It most likely has a multifactorial aetiology influenced by autoimmune, inflammatory, genetic, and possibly infective factors. PSC frequently co-exists with inflammatory bowel diseases—in particular, ulcerative colitis (UC) is present in up to 80% of PSC cases^1^. Persistent biliary tree damage leads to chronic cholestasis and episodes of cholangitis. PSC is also associated with greater risk of cholangiocarcinoma, which reportedly occurs in 10–12% of patients^2^.

The molecular mechanisms underlying the responses of liver and intestine tissue to chronic cholestasis in PSC remain largely unknown. To prevent intracellular accumulation of cytotoxic bile acids (BAs), specific plasma membrane transporters and nuclear receptors rigidly regulate BA transport and metabolism. Intestinal BA uptake directly and indirectly influences hepatic BA homeostasis, with both functions primarily regulated by farnesoid X receptor (FXR)^3^,^4^. FXR is mainly expressed in ileal enterocytes, but also in the liver and kidney. FXR’s predominant ligand is chenodeoxycholic acid (CDCA); other BAs also act as ligands but with lower efficacy. The main physiological role of FXR is to function as a BA sensor in enterohepatic tissues. FXR activation in enterocytes downregulates BA intestinal absorption and upregulates BA efflux pumps. This pathway involves the apical sodium-dependent bile acid transporter (ASBT; SLC10A1) and the heterodimeric organic solute transporters α and β (OSTα and OSTβ)^5^,^6^,^7^.

ASBT is expressed in the apical membrane of ileal enterocytes, and is critical for intestinal reabsorption of unconjugated bile acids. In contrast, OSTα and OSTβ expressions are positively regulated by BA-activated FXR and are largely restricted to the basal membrane of enterocytes. ASBT and OSTα/β also exist in cholangiocytes and in renal proximal tubule cells, where they promote bile acid reabsorption from bile ducts and blood circulation. FXR’s suppressing effects are facilitated by a small heterodimer partner (SHP) that lacks a DNA-binding domain and that competitively binds and negatively interacts with other transcriptional factors—such as liver receptor homolog-1 (LRH-1), hepatocyte nuclear factor-4α (HNF-4α), and retinoid X receptor (RXR)^8^,^9^. These factors bind to bile acid response elements (BAREs) located in the promoter regions of many genes, including *ASBT, CYP7A1*, and *CYP8B1*^10^.

Bile acids modulate their own synthesis through both intestinal and hepatic negative feedback. In the intestines, BA-activated FXR stimulates synthesis of the intestinal hormone fibroblast growth factor 19 (FGF19), which is secreted into the portal vein. In hepatocytes, BA-activated FXR triggers a FGF receptor 4 (FGFR4)-dependent signalling cascade and represses de novo BA synthesis by inhibiting cholesterol 7α-hydroxylase (CYP7A1). This enzyme is a rate-limiting factor in bile acid synthesis, initiating the production of two primary bile acids: CA and CDCA. Further CA synthesis requires microsomal sterol 12α-hydroxylase (CYP8B1), such that this enzyme’s activity determines the CA-to-CDCA ratio in the human liver. Over the course of sustained cholestasis, bile acids may activate other nuclear receptors, including the pregnane X receptor (PXR) or the constitutive androstane receptor (CAR) that triggers induction of the intestinal detoxification machinery. Every enterocyte contains the phase I hydroxylating enzyme CYP3A4, which metabolizes BA and protects enterocytes from accumulation of possibly harmful BAs.

Recent reports suggest a discrepancy between animal models and a type of cholestasis in humans with regards to the molecular mechanism of maintaining BA homeostasis. It remains unclear whether corresponding adaptive responses are coordinated in PSC-induced persistent cholestasis. Considering the practical limitations of human studies that have been unable to accurately determine the precise mechanism of cellular regulation of BA homeostasis, here we focus on assessing the molecular expressions of the factors in intestine and hepatic tissue of PSC patients.

## Results

### Gene expression patterns in different parts of the human intestine

**Figure 1:**
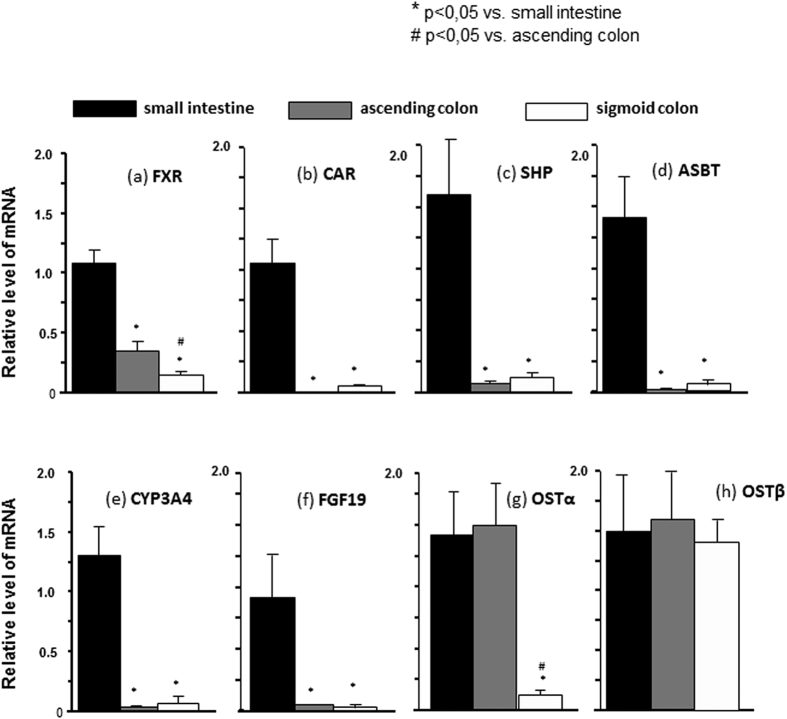
mRNA levels of the investigated genes in different gut segments compared to in the ileum. **(a–f)** mRNA expressions of FXR, CAR, SHP, ASBT, CYP3A4, and FGF19 were significantly higher in the ileum compared to in the colon. **(g)** OSTα expressions were similar between the ileum and the ascending colon, and significantly lower in the sigmoid colon. **(h)** OSTβ mRNA was uniformly expressed in all three examined parts of the intestine. mRNA expression levels are presented as fold-change relative to controls, and normalized relative to glyceraldehyde 3-phosphate dehydrogenase (GAPDH).

To examine the expression patterns of the assessed genes in the healthy human gut, we first assessed the mRNA levels in different gut segments compared to in the ileum (Fig. 1). The mRNA expressions of FXR, CAR, SHP, ASBT, CYP3A4, and FGF19 were significantly higher in the ileum compared to in the colon (Fig. 1a–f). In contrast, OSTα and OSTβ mRNA expressions were comparable between the intestine and colon. Similarly, OSTα mRNA expression was similar in the ileum and in the ascending colon (Fig. 1g), and OSTβ mRNA was equally expressed in all three examined parts of the intestine (Fig. 1h).

### FXR, SHP, and FGF19 expressions in the intestine

**Figure 2:**
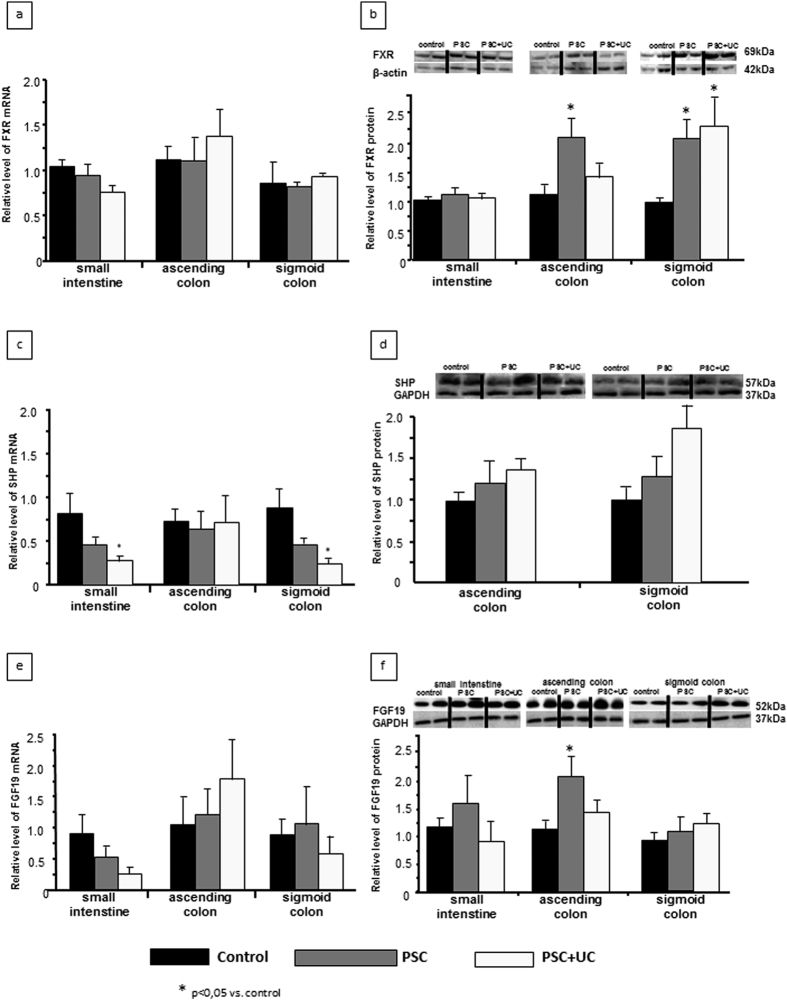
Intestinal expressions of FXR, SHP, and FGF19. Adaptive regulation of FXR expression was evaluated in three parts of the gut, with comparison between cases of cholestasis and control tissues. **(a)** FXR mRNA expression was comparable between the terminal ileum, ascending colon, and sigmoid colon in all groups. **(b)** FXR protein expression did not differ between PSC patients and controls in the ileum, but was significantly enhanced in PSC patients compared to control subjects in both the ascending and sigmoid colon. Within the PSC + UC group, this increased FXR protein expression was only detected in the sigmoid colon. **(c)** SHP mRNA expressions in the terminal ileum and the colon were equal among all examined groups, aside from a lower SHP mRNA level in the descending colon among PSC + UC subjects. **(d)** SHP protein levels were similar among all analysed samples regardless of the part of the gut. **(e)** FGF19 mRNA levels were similar in all examined parts of the gut, regardless of the examined group. **(f)** Intestinal FGF19 protein levels in PSC and PSC + UC patients were comparable to in controls, except for an elevated level in the ascending colon of PSC patients. mRNA expression levels are presented as fold-change relative to control, and were normalized relative to glyceraldehyde 3-phosphate dehydrogenase (GAPDH). Changes in protein levels were determined using densitometry analyses following normalization to GAPDH or β-actin as a loading control, and are presented as fold-change relative to control.

We next examined the adaptive regulation of FXR expression in three parts of the gut in cases of cholestasis, and compared these findings with the levels obtained in corresponding control tissues. In all groups, FXR mRNA was uniformly expressed in the terminal ileum, ascending colon, and sigmoid colon (Fig. 2a). The protein level of FXR was also unchanged in the ileum. However, FXR protein expression was significantly enhanced in the colon of PSC patients compared to control subjects, showing a 2-fold increase in both the ascending colon (2.1 ± 0.8 in PSC vs. 1.0 ± 0.3 in controls, *P* = 0.02) and sigmoid colon (2.01 ± 0.3 in PSC vs. 1.02 ± 0.1 in controls, *P* = 0.04; Fig. 2b). Within the PSC + UC group, this increased FXR protein expression was found only in the sigmoid colon (2.3 ± 0.5 in PSC + UC vs. 1.0 ± 0.1 in controls, *P* = 0.01; Fig. 2b).

The *SHP* gene was equally expressed in the terminal ileum and the colon in all examined groups. However, PSC + UC subjects showed reduced SHP mRNA expression in the descending colon (Fig. 2c). SHP protein levels were similar in all analysed samples and in all parts of the gut (Fig. 2d). As FGF19 synthesis is induced in enteric mucosa in response to FXR activation, we also evaluated the expression of this growth factor in the intestinal tissue. FGF19 mRNA levels were similar in all examined parts of the gut and in all examined groups (Fig. 2e). Moreover, intestinal FGF19 protein levels in both the PSC and PSC + UC groups were comparable to those in controls, except for a significant elevation in the ascending colon of PSC patients (Fig. 2f).

### Intestinal expressions of BA transporters and the detoxification enzyme CYP3A4

**Figure 3:**
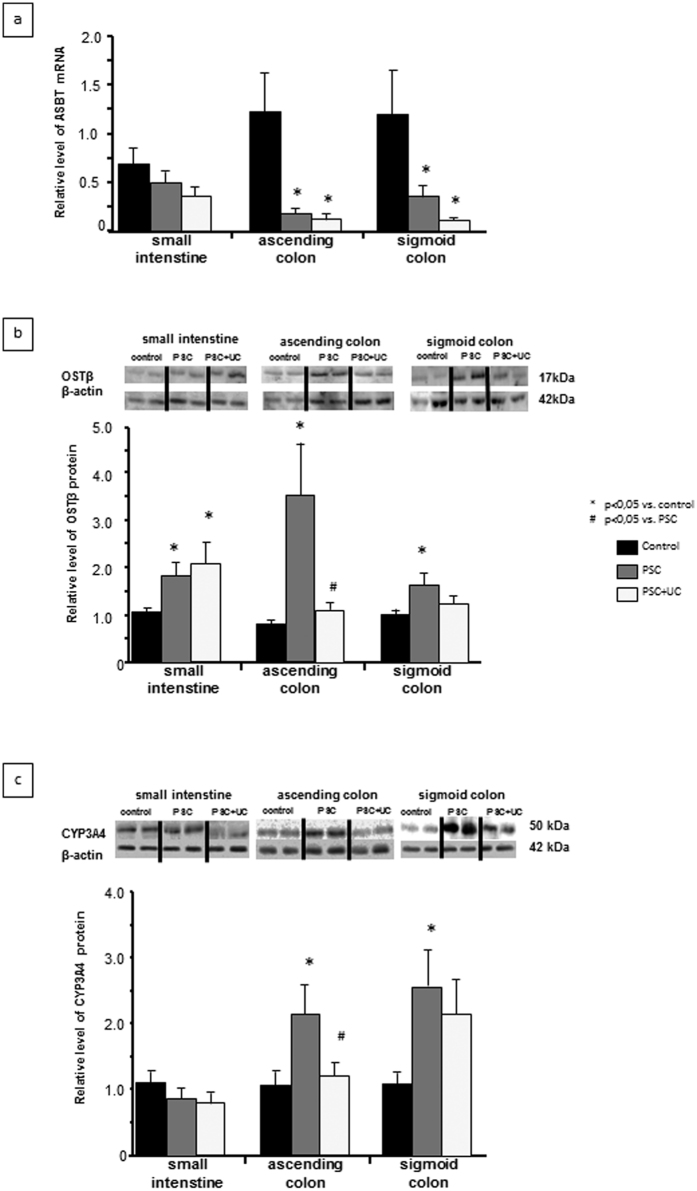
Intestinal expressions of bile acid transporters and the detoxification enzyme CYP3A4. **(a)** Compared to controls, the PSC and PSC + UC groups showed decreased ASBT mRNA expression in both the ascending and descending colon. **(b)** In PSC patients compared to controls, OSTβ protein levels were significantly increased in all three parts of gut. However, within the PSC + UC group, enhanced OSTβ protein expression was observed only in the terminal ileum. **(c)** PSC patients did not show altered CYP3A4 protein expression in the ileum; however, CYP3A4 protein expression was enhanced compared to controls in the ascending and sigmoid colon. Patients with PSC + UC showed unaltered CYP3A4 protein expression compared to in control tissue. mRNA expression levels were normalized relative to glyceraldehyde 3-phosphate dehydrogenase (GAPDH). Changes in protein levels were determined using densitometry analyses after normalization to β-actin as a loading control. Gene expression levels are presented as fold-change relative to control.

**Figure 4:**
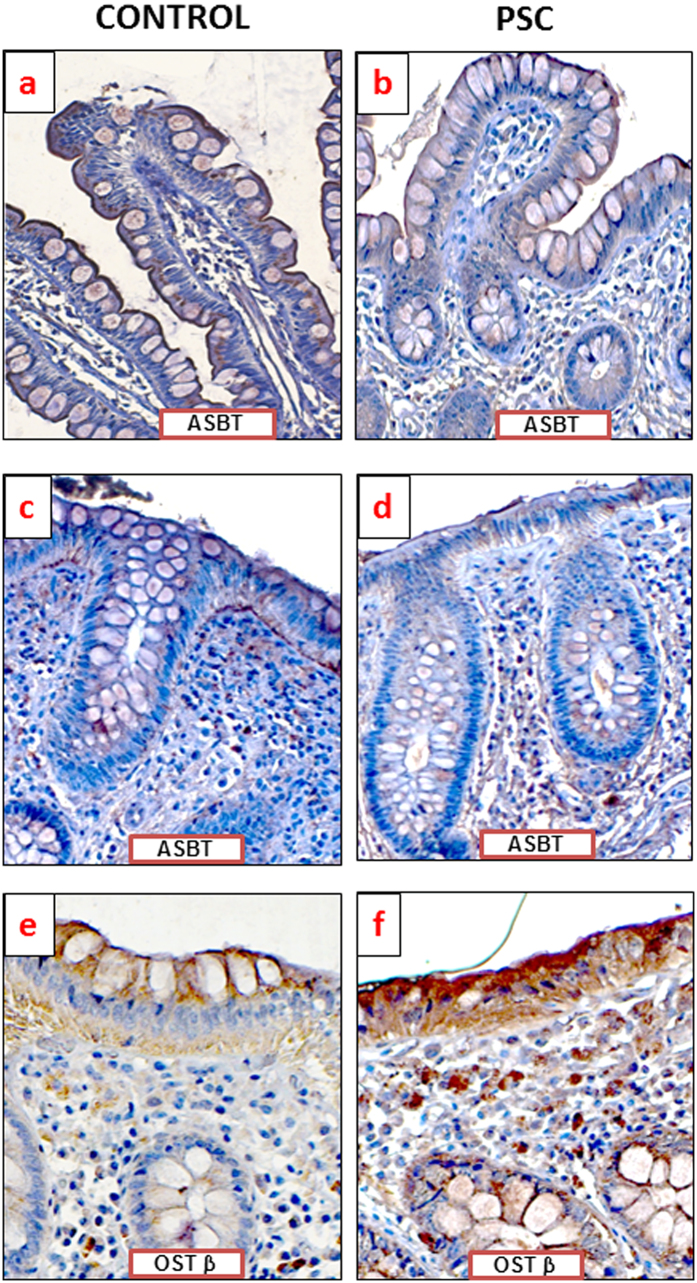
Immunohistochemical localization of apical sodium-dependent BA transporter (ASBT) and OSTβ proteins in human intestinal tissue. Representative photomicrographs show ASBT expression on the apical membrane of human ileum (**a,b**) or ascending colon (**c,d**) and OSTβ in human ascending colon (**e,f**). Brown positive staining of intestinal epithelium were demonstrated in cells of control subjects (**a,c,e**) and PSC patients (**b,d,f**) All pictures at 400-fold magnification.

Enhanced FXR expression impacts the balance between BA uptake and elimination; therefore, we examined the expressions of bile acid transporters in the intestine. In both the PSC and PSC + UC patient groups, the response to cholestasis involved decreased ASBT mRNA expression in the ascending colon (0.2 ± 0.1 vs. 1.2 ± 0.4, *P* = 0.02 for PSC vs. controls; 0.1 ± 0.1 vs. 1.2 ± 0.4, *P* = 0.02 for PSC + UC vs. controls) and descending colon (0.4 ± 0.1 vs. 1.2 ± 0.5, *P* = 0.04 for PSC vs. controls; 0.1 ± 0.03 vs. 1.2 ± 0.5; *P* = 0.03 for PSC + UC vs. controls) (Fig. 3a). In contrast, the protein level of OSTβ (BA transporter localized to the basal membrane of enterocytes) was significantly increased of in all three parts of the gut among PSC patients compared to controls, with a 1.8-fold increase in the ileum (*P* = 0.03), a 3.5-fold increase in the ascending colon (*P* = 0.007), and a 1.6-fold increase in the descending colon (*P* = 0.03) (Fig. 3b). However, within the PSC + UC group, enhanced OSTβ protein expression was observed only in the terminal ileum (2.2 fold vs. controls, *P* = 0.02). In agreement with molecular quantitative evaluations, immunohistochemical analyses of protein expression demonstrated lower intensity of ASBT staining in human ileum and ascending colon of patients with PSC (Fig. 4b,d), whereas OSTβ protein expression was more pronounced in intestinal epithelium cells of PSC patients (Fig. 4e,f).

CYP3A4 protein expression was not changed in the ileum, but synthesis of this enzyme was significantly increased in PSC patients compared to controls in the ascending colon (2.2 ± 0.5 vs. 1.1 ± 0.2; *P* = 0.017) and sigmoid colon (2.6 ± 0.5 vs. 1.1 ± 0.2, *P* = 0.02) (Fig. 3c). Moreover, the manifestation of inflammation in the colon tissue of PSC + UC patients abrogated this increased CYP3A4 expression (Fig. 3c). Furthermore, the CYP3A4 protein level positively correlated with FXR protein expression (R = 0.59; Z-value = 5.8; *P* < 0.0001).

### Hepatic expressions of adaptive response genes and relevant nuclear receptors

**Figure 5:**
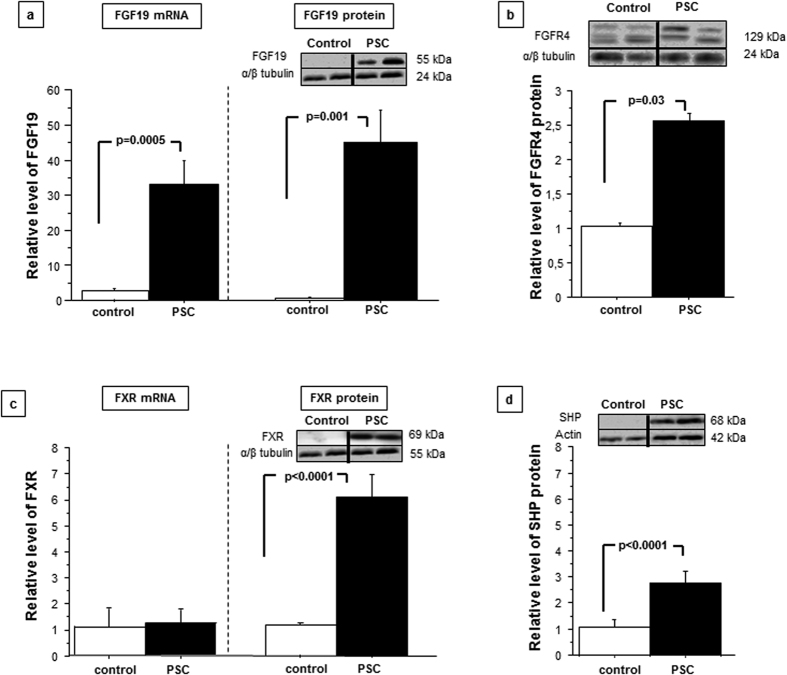
Hepatic expressions of adaptive response genes and relevant nuclear receptors. **(a)** In the liver tissue, FGF19 mRNA and protein levels were significantly increased in PSC patients compared to in control tissue. **(b)** FGFR4 protein expression was enhanced in the liver tissue of patients with PSC compared to that of controls. **(c)** FXR protein levels were considerably increased in patients with PSC compared to controls **(d)**, which was accompanied by enhanced SHP protein expression. mRNA expression levels were normalized relative to glyceraldehyde 3-phosphate dehydrogenase (GAPDH). Changes in protein levels were determined using densitometry analyses after normalization to α/β-tubulin or actin as a loading control. Gene expression levels are presented as fold-change relative to control.

**Figure 6:**
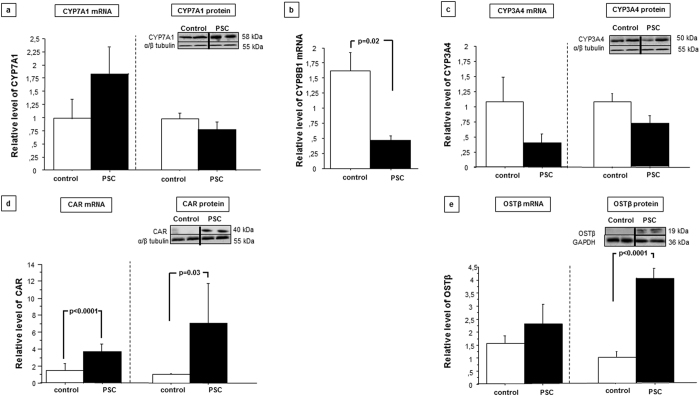
Hepatic expressions of adaptive response genes and relevant nuclear receptors. **(a)** Hepatic CYP7A1 protein levels were similar in PSC patients and in control tissue. **(b)** CYP8B1 protein expression was considerably reduced in patients with PSC vs. controls. **(c)** Hepatic CYP3A4 protein expression was unchanged in PSC patients compared to in control tissue. Compared to control tissue, PSC patient samples showed substantially enhanced protein expressions of the nuclear receptor CAR **(d)**. **(e)** Hepatic OSTβ mRNA expression was unchanged in patients with PSC compared to controls. Changes in protein levels were determined using densitometry analyses after normalization relative to α/β tubulin as a loading control. mRNA expression levels were normalized relative to glyceraldehyde 3-phosphate dehydrogenase (GAPDH). Gene expression levels are presented as fold-change relative to control.

Since intestinal FGF19 reportedly influences hepatic bile acid synthesis, we further investigated the hepatic expressions of the examined factors. Within the liver tissue of PSC patients, FGF19 mRNA and protein levels were sharply increased (33-fold *P* = 0.0005 and 56-fold, *P* = 0.001 vs. controls, respectively; Fig. 5a). Protein expression of FGFR4 was also enhanced in PSC patients compared to controls, but to a much lesser extent (2.55 ± 0.1 vs. 1.03 ± 0.5, *P* = 0.03; Fig. 5b). In terms of FXR expression in cirrhotic PSC livers, the level of mRNA was comparable to controls, however the protein level was substantially increased (5-fold, *P* < 0.0001 vs. controls) which was accompanied by enhanced SHP expression (2.88 ± 0.1 vs. 1.05 ± 0.8, *P* < 0.0001 vs. control; Fig. 5c,d). Western blot and real-time PCR analyses demonstrated that hepatic CYP7A1 mRNA and protein levels were not reduced in patients with PSC, regardless of the presence of activated FXR (1.7 ± 0.5 in PSC vs. 1.03 ± 0.3, and 0.75 ± 0.1 in PSC vs. 0.96 ± 0.1 in controls, respectively non-significant difference; Fig. 6a). Seeing that this phenomenon could have a potential effect on bile acids synthesis from cholesterol we have performed an additional analysis using our clinical database. We looked at cholesterol, alkaline phosphatase (ALP) and bilirubin levels at the diagnosis in 155 consecutive patients with PSC. As we previously demonstrated that CYP7A1 protein was reduced in response to activation of FXR in patients with primary biliary cholangitis (PBC)^11^ we have used this group, comprising of 266 patients as controls. Indeed, we have found a significantly lower levels of cholesterol (normal values 120–200 mg/dl) in patients with PSC (222 ± 91 mg/dl in PSC vs 247 ± 107 mg/dl in PBC; p = 0.016) despite the fact that they were more cholestatic biochemically with mean ALP (normal values 30–120U/l) of 440 ± 347U/l as compared to 363 ± 295U/l in PBC group (p = 0.017.) Mean bilirubin levels were comparable in both groups.

Conversely, gene expression of CYP8B1 (another FXR-dependent enzyme) was reduced by 64% in patients with PSC compared to controls (*P* = 0.02; Fig. 6b). We did not observe the enhanced expression of hepatic CYP3A4 in PSC patients neither on mRNA nor protein level (Fig. 6c), whereas the expression of nuclear receptors that induce CYP3A4 transcription, including PXR^12^ and CAR were substantially increased both on mRNA and protein levels. (3.09 ± 0,3 vs. 1.4 ± 0.3, and 7.1 ± 4 vs. 1.03 ± 0.2, P = <0.001 and *P* = 0.03 vs. controls, respectively Fig. 6d). Notably, we detected great inter-individual variability of CYP3A4 mRNA expression within both the control and PSC groups. To prevent bile acid accumulation in hepatocytes, FXR promotes alternative efflux of bile acids into the sinusoidal blood via OSTα/β transporters. We observed no increase in hepatic OSTβ gene expression among PSC patients, yet the protein level of OSTβ were notably increased (4.2 ± 0.08 in PSC vs. 1.0 ± 0,03 in controls, P < 0.0001 vs. controls (Fig. 6e).

## Discussion

Hepatic and intestinal responses to chronic cholestasis have predominantly been studied in various animal models, with relatively scarce data from human studies. Our present study involved an in-depth analysis of the intestinal and hepatic responses to PSC-induced chronic cholestasis. An important novel aspect of the current study is the finding that adaptive responses which ensure hepatic BA homeostasis are impaired in sustained cholestasis triggered by PSC. Unlike in our previous study on patients with primary biliary cholangitis (PBC)^11^ we detected no inhibition of CYP7A1 expression, which is a key mechanism involved in reducing hepatic BA synthesis. Simultaneously, we observed a significantly lower levels of cholesterol in patients with PSC when compared to patients with PBC what could be an indirect evidence of this molecular phenomenon. Moreover, despite the cholestasis and strong activation of nuclear receptors, we found no enhancement of the expression of the detoxification enzyme CYP3A4. Molecular analyses of human biopsies from different gut segments revealed no changes in the ileum. Rather, all observed molecular alterations were detected further down the intestinal tract, in colonic tissue. Moreover, expression of the apical BA influx transporter ASBT was inhibited by activated FXR, with corresponding enhancement of OSTβ protein expression in the ileum.

Bile acids, particularly chenodeoxycholic acid, can modulate the expressions of enzymes involved in their own synthesis by binding to FXR—thereby creating a negative feedback loop. In the present study, despite the enhanced expressions of hepatic FXR and nuclear repressor SHP, we detected no CYP7A1 inhibition. These findings are in agreement with the previous report by Kong *et al*. demonstrating that hepatic Fxr activation via Shp induction plays a less important role in suppressing hepatic Cyp7a1 expression compared to the ileal route^13^. On the other hand, CYP7A1 can also be regulated via some FXR- and SHP-independent pathways. For example, BAs can directly decrease activity of HNF4α, the factor that activates CYP7A1 transcription^9^. However, our present results showed the opposite effect, with substantially increased HNF4α protein levels in the examined liver tissue (data not shown). Thus, the lack of CYP7A1 repression cannot be due to BA-modulated inhibition of HNF4α gene transcription.

The terminal ileum profoundly impacts hepatic CYP7A1 gene regulation through the release of endocrine-acting FGF19^14^. In the liver, FGF19 binds to FGFR4, and represses CYP7A1 through a JNK-dependent pathway. Operative gut–liver signalling is considered crucial for BA homeostasis, and experiments in liver- and ileum-specific FXR-deficient mice suggest that CYP7A1 repression via the Fgf15 ileal pathway is dominant over hepatic negative feedback pathways^15^. Thus, under physiological conditions, intestinal FXR activation plays a major role in regulating BA synthesis. Experimentally stimulated intestinal Fxr transcription reduced the hepatic BA pool in mice with intrahepatic or extrahepatic cholestasis, in association with Fgf15 synthesis activation, leading to suppression of Cyp7a1 and Cyp8b1^16^. Notably, our present results showed no detectable alterations of FGF19 mRNA and protein levels in most intestinal samples from PSC and PSC + UC patients, except for a significant increase of FGF19 protein in the ascending colon of the PSC group. Thus our work provides novel observation as no prior studies have analysed FGF19 expression in the intestinal tissue of patients with cholestatic diseases and prior data come mostly from animal models of cholestasis. FGF19 expression was recently reported in other tissues, including the human gallbladder and the common bile duct, with only minor expression observed in the ileum^17^,^18^. However, indirect evidence for the harmful effects of the FXR-FGF19 axis interruption on the liver brings recent study on intestinal failure-associated liver disease (IFALD). In this condition short bowel syndrome results in low serum FGF19 levels and dysfunction of the FXR-FGF19 axis, which are considered as primary causes of disproportionate BA synthesis in the liver. Thus hepatobiliary complications seen in IFALD may arise from intrahepatic accumulation of toxic bile salts due to impaired FXR/FGF19-mediated liver repair^19^,^20^.

Our present findings suggest that mechanisms of hepatic BA homeostasis may be seriously impaired in PSC due to the lack of repression of CYP7A1, the rate-limiting enzyme for bile acid synthesis. Discrepancies between our results and previous observations may be due to the fact that much of the prior research in this field was performed in animal models of experimentally induced acute cholestasis, and thus the findings may not directly translate to humans. Moreover, sustained cholestasis induced by serious cholangiopathy could promote prolonged changes in the expressions of many different factors involved in BA homeostasis. Interestingly, the livers of PSC patients exhibited substantial induction of hepatic FGF19 mRNA and protein in association with the 2.5-fold increased expression of its receptor, FGFR4. These observations are intriguing since intestinal tissue has been considered the main reserve of this endocrine factor. It remains uncertain whether hepatic FGF19 contributes to regulation of bile salt synthesis under cholestatic conditions.

A prior study in patients with pancreatic or periampullary malignancy examined adaptive changes in the liver under conditions of extrahepatic cholestasis, and reported elevated hepatic FGF19 mRNA levels in patients with extrahepatic cholestasis, which returned to control values after 12 weeks of biliary stent application^21^. Those results revealed the liver to be an additional FGF19 source. However, the examined conditions might not represent the intrahepatic cholestasis found in chronic cholestatic conditions, such as PSC, since they did not induce expression of the nuclear receptors (e.g. CAR, FXR, PXR, and SHP) that are activated by accumulated bile acids^21^. Our present findings revealed high levels of the endocrine factor FGF19 in the livers of PSC patients, which is a phenomenon that we previously observed in PBC^11^. These findings may suggest that during the course of sustained cholestasis, FGF19 triggers molecular changes in the liver through an autocrine signalling pathway. Interestingly, FGF19 synthesis and responsiveness (CYP7A1 inhibition) have been demonstrated in cultured hepatocytes and PBC, possibly suggesting that BA-activated hepatic FXR can induce FGF19 in hepatocytes to inhibit CYP7A1 via an autocrine/paracrine mechanism^22^,^23^.

Our present study also demonstrated that the lack of CYP7A1 repression was accompanied by significantly reduced CYP8B1 mRNA levels in the livers of patients with PSC, where the FXR and SHP genes were activated. This inconsistency in the adaptive response to hepatic BA accumulation may be explained by observations in animal models showing that Shp does not substantially contribute to Cyp7a1 inhibition (~15%), but is as important as Fgf15 in suppressing Cyp8b1 gene expression^13^. Further supporting this concept, a prior study showed that Shp reduction did not impact Cyp7a1 gene expression, but rather markedly reduced Cyp8b1 gene expression^24^,^25^.

Activation of another nuclear factor, PXR regulates bile acid detoxification via CYP3A4, SULT2A1, and UGT2B4, thus protecting the cells from BA-induced damage in both the liver and ileum^26^,^27^,^28^. CYP3A4 plays an important role in detoxifying bile acids by catalysing their hydroxylation and thereby increasing bile acid hydrophilicity and decreasing their toxicity. Our present results suggest that this hepatic detoxification ability may be impaired by an insufficient increase of hepatic CYP3A4 expression in PSC. This change could be specific to PSC, as the cholestatic condition primary biliary cirrhosis (PBC) is associated with substantially increased CYP3A4 protein levels^29^. Cholestatic rodent livers have shown induction of the detoxification enzymes Cyp3a11 and soluble sulfotransferases (Sult2a1), which contribute to decreasing bile acid toxicity^30^,^31^. The molecular mechanism of adaptive response may vary between species and according to the type of cholestasis.

Only limited data are available regarding CYP3A4 expression in cholestatic livers. The presently reported lack of detectable changes in CYP3A4 protein levels between controls and PSC donors is consistent with a previous report (O. Barbier personal communication). In contrast, another study reported substantially reduced CYP3A4 expression in liver samples of patients with gallstone-related biliary obstruction^32^. Notably, Zollner and co-workers did not detect increased CYP3A4 mRNA levels in cirrhotic livers of patients with PBC^33^, while we recently observed significantly increased CYP3A4 protein expression in PBC patients^29^. This inconsistency could be related to the fact that our present analysis was based on protein levels, and thus could more accurately detect the influence of post-transcriptional changes. Zollner and co-workers described unchanged mRNA expression of this detoxification enzyme in patients with PBC, which they explained based on the relatively low expressions of hepatic FXR, PXR, and CAR mRNA. However, in our present and prior studies, we found that the protein levels of these nuclear receptor were substantially enhanced in both PSC and PBC^12^,^23^. The authors also suggested an alternative explanation based on the assumption that the NR-induced CYP3A4 expression is dependent on the disease stage, with CYP3A4 induction present only in the early disease stages. However, the changes in our present study were detected in the cirrhotic stage of the both PSC and PBC.

We found that protein expression of the detoxification enzyme CYP3A4 was substantially induced in both the ascending and sigmoid colon, but not in the ileum. These observations suggest that the FXR-induced adaptive response was extended to further portions of the gut to protect the mucosa of PSC patients against enhanced toxic BA accumulation. Bile acid accumulation plays a critical role in cholestasis pathophysiology. FXR is instrumental in the coordinated regulation of hepatic defence against bile acids via induction of alternative export. Indeed, our molecular analyses revealed enhanced expressions of the basolateral FXR-dependent bile acid efflux transporter, OSTβ in livers and intestines of PSC patients. These results are in agreement with the observations reported in two cholestatic conditions such as experimental cholestasis induced by common bile duct ligation, and in human livers of patients with PBC^7^. In PSC colon we detected substantial repression of the influx transporter, with correspondingly increased protein expression of the basolateral transporter OSTβ. Reduced levels of ASTB mRNA in all three parts of the intestine in PSC patients were not accompanied by changes in SHP mRNA, potentially suggesting the involvement of another regulatory pathway. ASBT expression is negatively regulated by a number of mechanisms. In addition to the SHP-dependent route, a second inhibitory pathway has been described that involves the activator protein-1 (AP-1) c-Fos^34^. Notably, FGF-19 was recently shown to repress ASBT expression in the ileum in a autocrine manner via the signalling molecule c-Fos^35^.

Our study has several obvious limitations, including the fact that two separate groups of patients were analysed. However, it would be extremely difficult to simultaneously obtain both explanted liver and colonic tissues from the same patient. Despite the limitations, our present findings could be of value in further elucidation of the mechanisms involved in pathogenesis of chronic cholestatic conditions. PBC and PSC undeniably show important differences in terms of hepatic nuclear receptors, enzymes, and transporter expressions, as has been previously demonstrated by our group and others^12^,^29^,^36^. The current results highlight the need for further investigations of the underlying processes leading to the lack of CYP7A1 suppression and the impaired CYP3A4 induction that were observed in PSC but not PBC.

## Methods

### Study Design

We conducted a two-part study comparing findings in enteric mucosal biopsy specimens from PSC patients with or without colitis, and in liver tissue samples from PSC patients who underwent liver transplantation. The research protocol was approved by the Ethics Committee of Pomeranian Medical University and conformed to the ethical guidelines of the 1975 Declaration of Helsinki.

### Patients Characteristics

The first part of the study enrolled 22 PSC patients who underwent routine surveillance colonoscopies. These patients were divided into two groups: the PSC group included 11 patients who had never been diagnosed with inflammatory bowel disease (9 males, 2 females; mean age, 30 ± 9 years), and the PSC + UC group included 11 patients showing macroscopic features of ulcerative colitis (UC; 8 males, 3 females; mean age, 35 ± 17 years). Clinical data were collected from cases and controls prior to colonoscopy and tissue sampling. All PSC patients were treated with ursodeoxycholic acid (average dose, 15 mg/kg bw). Patients with UC additionally received 5ASA (2–3 g/daily). For molecular analysis, we obtained three biopsy specimens per patient from the gut, including the terminal ileum, ascending colon, and sigmoid colon. Although secondary BAs produced by bacteria in the colon are thought to enter the colonic enterocyte entirely by passive diffusion, we also investigated BA transporter expressions in the colons of PSC patients to examine for any changes that could facilitate BA translocation. The control group for this part of the study comprised 14 subjects (8 males, 6 females; mean age, 50 ± 16 years) who underwent colonoscopies for various indications and showed no macroscopic changes in their colons.

In the second part of this study, liver tissue specimens were collected from explanted livers of 11 PSC patients who underwent liver transplantation. Control liver tissue samples (n = 19) were obtained from large-margin liver resections of colorectal metastases that showed no pathologist-identified microscopic changes of liver disease. Each patient gave informed consent prior to participating in this study. Table 1 presents patient demographic details.

### Tissue specimen preparation

In preparation for future analyses, intestine tissue biopsies were either (i) stored in RNAlater for subsequent analysis of mRNA expression (AM7021; Applied Biosystems, Carlsbad, CA, USA), (ii) fixed in neutral-buffered formalin for histological assessment, or (iii) immediately frozen in liquid nitrogen for proteomic analyses. Histological assessment was performed by a pathologist (EU) who was blinded to patient clinical and laboratory data, utilizing the histological grading scale introduced by Geboes *et al*.^37^. Six histological features were evaluated as previously described^12^. Due to the limited available biopsy material, distinct investigations were performed based on mRNA analysis. However key findings were validated at the protein level.

Liver tissue specimens were collected as tissue blocks (∼1 cm^3^), which were immediately frozen in liquid nitrogen and stored at −75 °C until use. For analysis, samples were powdered and homogenized in an appropriate lysis buffer to extract either total RNA or protein.

### Quantitative real-time PCR (qRT-PCR) analysis

Total RNA was extracted from 50-mg tissue samples using the RNeasy Mini Kit (Qiagen). Isolated RNA quantity and quality was determined using an Epoch spectrophotometer (BioTek). Subsequently, cDNA was prepared from 50 ng of total cellular RNA in an 20-μL reaction volume, using the SuperScript^TM^ First-Strand Synthesis System for RT-PCR (Invitrogen). Quantitative real-time PCR was performed using the 7500 Fast Real-Time PCR System (Applied Biosystems, USA), with the following pre-validated Taqman gene expression assays: FXR (NR1H4), Hs00231968_m1; CAR (NR1I3), Hs00901571_m1; FGF19, Hs00192780_m1; CYP8B1, Hs00244754_s1; CYP3A4, Hs00604506_m1; CYP7A1, Hs00167982_m1; SHP(NR0B2), Hs00222677_m1; ASBT(SLC10A2), Hs01001553_m1; OSTalpha, Hs00380895_m1; OSTbeta, Hs01057182_m1; GAPDH, Hs99999905_m1; 18S, Hs99999901_s1. Each 20-μL reaction mix included TaqMan Gene Expression Master Mix (Applied Biosystems, USA) and 2 μL of cDNA. Each sample was analysed simultaneously in two replicates Calculations were performed using the ΔΔCt relative quantification method. The Ct values for each sample were normalized to the mean value obtained for two endogenous control genes: glyceraldehyde-3-phosphate dehydrogenase (GAPDH) and 18S ribosomal RNA.

### Western blot analysis

Frozen liver tissues were lysed in a RIPA buffer supplemented with cOmplete™ Mini Protease Inhibitor Cocktail (Roche) and phosphatase inhibitors (PhosSTOP EASYpack; Roche). Extracted proteins (60 μg) were resolved in SDS polyacrylamide gels, and then transferred onto a PVDF membrane using semi-dry transfer (Immobilon-P; Millipore). The following primary antibodies were used: anti-FGF19 (MAB969, R&D Systems; ab154185, Abcam), anti-FGFR4 (8562, Cell Signalling), anti-CYP7A1 (sc-25536, Santa Cruz), anti-CYP3A4 (sc-53850, Santa Cruz), anti-SHP (sc-15283, Santa Cruz), anti-FXR (sc-1204, Santa Cruz), anti-CAR (PP-N4111-00, R&D), and anti-OSTβ (sc-163192, Santa Cruz). Protein loading was normalized to GAPDH (sc-25778 + HRP; Santa Cruz), β-actin (sc-47778, Santa Cruz), and α/β-tubulin (#2148, Cell Signaling). The bands were visualized using the SuperSignal West Pico Chemiluminescent Detection System (Thermo Scientific). Image and densitometry analyses were performed using MicroChemi Imaging Systems and GelQuant software (DNR Bio-Imaging, Israel).

### Immunohistochemistry

After deparaffinization and antigen unmasking with citrate buffer (pH6.0 for 30 min in 98 °C) the sections were fixated in cold acetone for 5 minutes (−20 °C). Then the endogenous peroxide activity was blocked by the incubation in 3% methanolic hydrogen peroxide (30 minutes) followed by blocking of unspecific binding by Normal Horse Serum (Vector Laboratories, Burlingame, CA, USA). After washing in phosphate-buffered saline anti-OSTβ (sc-163192, Santa Cruz) and anti-ASBT (sc-27493, Santa Cruz) were used as primary antibodies and biotynaleted anti-mouse/anti-rabbit IgG (BA-1400, Vector Laboratories) served as secondary antibody. Reactions were visualized using ABC Vectastain and DAB kits (Dako, Agilent Technologies, Denmark). Additionally, tissue structures were visualized by Mayer’s Hematoxylin staining (DAKO). The negative controls, in which the primary antibodies were omitted, were included in the study and uniformly demonstrated no reaction. Images were acquired with ZEISS Axio Imager Z2 microscope equipped with Zen Pro 2011acquisition program.

### Statistical analyses

Data are presented as mean ± standard error (SD) unless otherwise indicated. All statistical analyses were performed using StatView-5 Software (SAS Institute, Cary, NC, US). Between-group differences were evaluated using non-parametric tests (Mann-Whitney U or Kruskal-Wallis) or analysis of variance (ANOVA) with Fisher’s PLSD. Correlations were calculated as Spearman’s rank correlation coefficients. A *P* value of 0.05 was considered to indicate statistical significance.

## Additional Information

**How to cite this article**: Milkiewicz, M. *et al*. Impaired Hepatic Adaptation to Chronic Cholestasis induced by Primary Sclerosing Cholangitis. *Sci. Rep.* **6**, 39573; doi: 10.1038/srep39573 (2016).

**Publisher's note:** Springer Nature remains neutral with regard to jurisdictional claims in published maps and institutional affiliations.

## Figures and Tables

**Table 1:** Demographic and laboratory features of analysed subjects.

	Control group (n = 14)	PSC (n = 11)	PSC + UC (n = 11)	PSC vs. control group	PSC + UC vs. control group	PSC + UC vs. PSC
Gender (M/F)	8/6	9/2	8/3	NS	NS	NS
Age (mean ± SE)	50 ± 4	30 ± 3	35 ± 5	P = 0.001	P = 0.04	NS
Hb (mmol/l, mean ± SE)	—	13.5 ± 0.9	13.2 ± 0.6	—	—	NS
Bilirubin (μmol/l, mean ± SE)	—	10.1 ± 1.6	25.3 ± 11.9	—	—	NS
ALP (U/l, mean ± SE)	—	159.4 ± 36.2	297.6 ± 81.7	—	—	NS
GGTP (U/l, mean ± SE)	—	302.4 ± 110.7	370.8 ± 190.3	—	—	NS
ALT (U/l, mean ± SE)	—	103.4 ± 30.7	61.4 ± 23.6	—	—	NS

Abbreviations: PSC- primary sclerosing cholangitis, UC – ulcerative colitis.

## References

[b1] Hirschfield G. M., Karlsen T. H., Lindor K. D. & Adams D. H. Primary sclerosing cholangitis. Lancet 382, 1587–1599 (2013). 23810223 10.1016/S0140-6736(13)60096-3

[b2] Milkiewicz P. & Wunsch E. Primary sclerosing cholangitis. Recent Results Cancer Res. 185, 117–133 (2011). 21822823 10.1007/978-3-642-03503-6_7

[b3] Makishima M. . Identification of a nuclear receptor for bile acids. Science 284, 1362–1365 (1999). 10334992 10.1126/science.284.5418.1362

[b4] Modica S., Gadaleta R. M. & Moschetta A. Deciphering the nuclear bile acid receptor FXR paradigm. Nucl. Recept. Signal. 8, e005 (2010). 21383957 10.1621/nrs.08005 PMC3049226

[b5] Ballatori N. . OSTalpha-OSTbeta: a major basolateral bile acid and steroid transporter in human intestinal, renal, and biliary epithelia. Hepatology 42, 1270–1279 (2005). 16317684 10.1002/hep.20961

[b6] Dawson P. A. . The heteromeric organic solute transporter alpha-beta, Ostalpha-Ostbeta, is an ileal basolateral bile acid transporter. J. Biol. Chem. 280, 6960–6968 (2005). 15563450 10.1074/jbc.M412752200 PMC1224727

[b7] Boyer J. L. . Upregulation of a basolateral FXR-dependent bile acid efflux transporter OSTalpha-OSTbeta in cholestasis in humans and rodents. Am. J Physiol Gastrointest. Liver Physiol 290, G1124–G1130 (2006). 16423920 10.1152/ajpgi.00539.2005

[b8] Neimark E., Chen F., Li X. & Shneider B. L. Bile acid-induced negative feedback regulation of the human ileal bile acid transporter. Hepatology 40, 149–156 (2004). 15239098 10.1002/hep.20295

[b9] Kir S., Zhang Y., Gerard R. D., Kliewer S. A. & Mangelsdorf D. J. Nuclear receptors HNF4alpha and LRH-1 cooperate in regulating Cyp7a1 *in vivo*. J. Biol. Chem. 287, 41334–41341 (2012). 23038264 10.1074/jbc.M112.421834 PMC3510831

[b10] Goodwin B. . A regulatory cascade of the nuclear receptors FXR, SHP-1, and LRH-1 represses bile acid biosynthesis. Mol. Cell 6, 517–526 (2000). 11030332 10.1016/s1097-2765(00)00051-4

[b11] Wunsch E. . Expression of hepatic Fibroblast Growth Factor 19 is enhanced in Primary Biliary Cirrhosis and correlates with severity of the disease. Sci. Rep. 5, 13462 (2015). 26293907 10.1038/srep13462 PMC4544021

[b12] Wunsch E. . Liver Expression of Sulphotransferase 2A1 Enzyme Is Impaired in Patients with Primary Sclerosing Cholangitis: Lack of the Response to Enhanced Expression of PXR. J Immunol. Res. 2015, 571353 (2015). 26504856 10.1155/2015/571353 PMC4609469

[b13] Kong B. . Mechanism of tissue-specific farnesoid X receptor in suppressing the expression of genes in bile-acid synthesis in mice. Hepatology 56, 1034–1043 (2012). 22467244 10.1002/hep.25740 PMC3390456

[b14] Inagaki T. . Fibroblast growth factor 15 functions as an enterohepatic signal to regulate bile acid homeostasis. Cell Metab 2, 217–225 (2005). 16213224 10.1016/j.cmet.2005.09.001

[b15] Kim I. . Differential regulation of bile acid homeostasis by the farnesoid X receptor in liver and intestine. J Lipid Res. 48, 2664–2672 (2007). 17720959 10.1194/jlr.M700330-JLR200

[b16] Modica S. . Selective activation of nuclear bile acid receptor FXR in the intestine protects mice against cholestasis. Gastroenterology 142, 355–365 (2012). 22057115 10.1053/j.gastro.2011.10.028

[b17] Zweers S. J. . The human gallbladder secretes fibroblast growth factor 19 into bile: towards defining the role of fibroblast growth factor 19 in the enterobiliary tract. Hepatology 55, 575–583 (2012). 21953282 10.1002/hep.24702

[b18] Zweers S. J. . Elevated interleukin-8 in bile of patients with primary sclerosing cholangitis. Liver Int. (2016). 10.1111/liv.13092 26866350

[b19] Mutanen A., Lohi J., Heikkila P., Jalanko H. & Pakarinen M. P. Loss of ileum decreases serum fibroblast growth factor 19 in relation to liver inflammation and fibrosis in pediatric onset intestinal failure. J Hepatol. 62, 1391–1397 (2015). 25595885 10.1016/j.jhep.2015.01.004

[b20] Walters J. R. . A new mechanism for bile acid diarrhea: defective feedback inhibition of bile acid biosynthesis. Clin. Gastroenterol. Hepatol. 7, 1189–1194 (2009). 19426836 10.1016/j.cgh.2009.04.024

[b21] Schaap F. G., van der Gaag N. A., Gouma D. J. & Jansen P. L. High expression of the bile salt-homeostatic hormone fibroblast growth factor 19 in the liver of patients with extrahepatic cholestasis. Hepatology 49, 1228–1235 (2009). 19185005 10.1002/hep.22771

[b22] Song K. H., Li T., Owsley E., Strom S. & Chiang J. Y. Bile acids activate fibroblast growth factor 19 signaling in human hepatocytes to inhibit cholesterol 7alpha-hydroxylase gene expression. Hepatology 49, 297–305 (2009). 19085950 10.1002/hep.22627 PMC2614454

[b23] Wunsch E. . Prospective evaluation of ursodeoxycholic acid withdrawal in patients with primary sclerosing cholangitis. Hepatology 60, 931–940 (2014). 24519384 10.1002/hep.27074

[b24] Mataki C. . Compromised intestinal lipid absorption in mice with a liver-specific deficiency of liver receptor homolog 1. Mol. Cell Biol. 27, 8330–8339 (2007). 17908794 10.1128/MCB.00852-07 PMC2169191

[b25] Lee Y. K. . Liver receptor homolog-1 regulates bile acid homeostasis but is not essential for feedback regulation of bile acid synthesis. Mol. Endocrinol. 22, 1345–1356 (2008). 18323469 10.1210/me.2007-0565 PMC2409274

[b26] Mouly S. . Hepatic but not intestinal CYP3A4 displays dose-dependent induction by efavirenz in humans. Clin. Pharmacol. Ther. 72, 1–9 (2002). 12151999 10.1067/mcp.2002.124519

[b27] Tirona R. G. . The orphan nuclear receptor HNF4alpha determines PXR- and CAR-mediated xenobiotic induction of CYP3A4. Nat. Med. 9, 220–224 (2003). 12514743 10.1038/nm815

[b28] Luo G. . CYP3A4 induction by drugs: correlation between a pregnane X receptor reporter gene assay and CYP3A4 expression in human hepatocytes. Drug Metab Dispos. 30, 795–804 (2002). 12065438 10.1124/dmd.30.7.795

[b29] Milkiewicz M. . Impaired expression of enzymes responsible for bile acid synthesis and detoxification in patients with primary sclerosinng cholangitis. J. Hepatology 62, S791–S792 (2015).

[b30] Bodin K., Lindbom U. & Diczfalusy U. Novel pathways of bile acid metabolism involving CYP3A4. Biochim. Biophys. Acta 1687, 84–93 (2005). 15708356 10.1016/j.bbalip.2004.11.003

[b31] Claudel T., Zollner G., Wagner M. & Trauner M. Role of nuclear receptors for bile acid metabolism, bile secretion, cholestasis, and gallstone disease. Biochim. Biophys. Acta 1812, 867–878 (2011). 21194565 10.1016/j.bbadis.2010.12.021

[b32] Chai J. . Hepatic expression of detoxification enzymes is decreased in human obstructive cholestasis due to gallstone biliary obstruction. PLoS. One. 10, e0120055 (2015). 25798860 10.1371/journal.pone.0120055 PMC4370735

[b33] Zollner G. . Expression of bile acid synthesis and detoxification enzymes and the alternative bile acid efflux pump MRP4 in patients with primary biliary cirrhosis. Liver Int. 27, 920–929 (2007). 17696930 10.1111/j.1478-3231.2007.01506.x

[b34] Chen F., Ma L., Al-Ansari N. & Shneider B. The role of AP-1 in the transcriptional regulation of the rat apical sodium-dependent bile acid transporter. J. Biol. Chem. 276, 38703–38714 (2001). 11509565 10.1074/jbc.M104511200

[b35] Ghosh A., Chen F., Banerjee S., Xu M. & Shneider B. L. c-Fos mediates repression of the apical sodium-dependent bile acid transporter by fibroblast growth factor-19 in mice. Am. J Physiol Gastrointest. Liver Physiol 306, G163–G171 (2014). 24309182 10.1152/ajpgi.00276.2013 PMC3920077

[b36] Bell L. N., Wulff J., Comerford M., Vuppalanchi R. & Chalasani N. Serum metabolic signatures of primary biliary cirrhosis and primary sclerosing cholangitis. Liver Int. 35, 263–274 (2015). 25181933 10.1111/liv.12680 PMC4293304

[b37] Geboes K. . A reproducible grading scale for histological assessment of inflammation in ulcerative colitis. Gut 47, 404–409 (2000). 10940279 10.1136/gut.47.3.404 PMC1728046

